# Complementary and alternative medicine for treatment of atopic eczema in children under 14 years old: a systematic review and meta-analysis of randomized controlled trials

**DOI:** 10.1186/s12906-018-2306-6

**Published:** 2018-09-26

**Authors:** Chun-li Lu, Xue-han Liu, Trine Stub, Agnete E. Kristoffersen, Shi-bing Liang, Xiao Wang, Xue Bai, Arne Johan Norheim, Frauke Musial, Terje Araek, Vinjar Fonnebo, Jian-ping Liu

**Affiliations:** 1 Centre for Evidence-Based Chinese Medicine, Beijing University of Chinese Medicine, Beijing, 100029 China; 2 The National Research Center in Complementary and Alternative Medicine (NAFKAM), Department of Community Medicine, Faculty of Health Science, UiT, The Arctic University of Norway, 9037 Tromsø, Norway; 3 School of Basic Medicine, Shanxi University of Chinese Medicine, Taiyuan, 030000 China

**Keywords:** Complementary and alternative medicine, CAM, Atopic eczema, Children, Randomized controlled trials, Systematic review, Meta-analysis, Clinical evidence

## Abstract

**Background:**

Due to limitations of conventional medicine for atopic eczema (AE), complementary and alternative medicine (CAM) is widely used as an alternative, maintaining, or simultaneous treatment for AE. We aimed to evaluate the beneficial and harmful effects of CAM for children with AE under 14 years old.

**Methods:**

We searched for randomized trials on CAM in 12 Chinese and English databases from their inception to May 2018. We included children (< 14 years) diagnosed with AE, who received CAM therapy alone or combined with conventional medicine. We extracted data, and used the Cochrane “Risk of bias” tool to assess methodological quality. Effect was presented as relative risk (RR) or mean difference (MD) with 95% confidence interval (CI) using RevMan 5.3.

**Results:**

Twenty-four randomized controlled trials involving 2233 children with AE were included. Methodological quality was of unclear or high risk of bias in general. The trials tested 5 different types of CAM therapies, including probiotics, diet, biofilm, borage oil, and swimming. Compared to placebo, probiotics showed improved effect for the SCORAD index (MD 9.01, 95% CI 7.12–10.90; *n* = 5). For symptoms and signs such as itching, skin lesions, CAM combined with usual care was more effective for symptom relief ≥95% (RR 1.47, 95% CI 1.30–1.68; *n* = 8), and for ≥50% symptoms improvement (RR 1.34, 1.25–1.45; *n* = 9) compared to usual care. There was no statistic significant difference between CAM and usual care on ≥95% improvement or ≥ 50% improvement of symptoms. However, swimming, diet and biofilm showed improvement of clinical symptoms compared with usual care. At follow-up of 8 weeks to 3 years, CAM alone or combined with usual care showed lower relapse rate (RR 0.38, 0.28–0.51, *n* = 2; RR 0.31, 0.24–0.40, *n* = 7; respectively) compared to usual care. Twelve out of 24 trials reported no occurrence of severe adverse events.

**Conclusions:**

Low evidence demonstrates that some CAM modalities may improve symptoms of childhood AE and reduce relapse rate. Safety remains unclear due to insufficient reporting. Further well-designed randomized trials are needed to confirm the potential beneficial effect and to establish safety use.

**Electronic supplementary material:**

The online version of this article (10.1186/s12906-018-2306-6) contains supplementary material, which is available to authorized users.

## Background

Eczema, as defined by the World Allergy Organization, encompasses both atopic and non-atopic conditions, and is commonly referred to as atopic eczema (AE) or atopic dermatitis (AD) [1]. AE is a chronically relapsing inflammatory skin disease, often found in children under the age of 14 years. It impairs people’s quality of life [2] and the prevalence of AE is estimated to be 15–20% in children worldwide [3]. As one of the most common inflammatory skin diseases, AE has a prevalence exceeding 10% of children in some populations [4]. There is an increasing number of studies focusing on AE, such as clinical trials and systematic reviews [5].

AE can be caused by multiple and complex risk factors such as irritants, contact allergens, food, inhaled allergens, stress or infection [6]. The pathogenesis of eczema is a complex interplay of numerous elements including immune, genetic, infection and neuroendocrine factors and their interaction with the environment [2]. Moreover, the diagnosis of AE relies on the assessment of clinical features because there is no definitive/conclusive test to diagnose the condition. The clinical characteristics are itching, skin inflammation, a skin barrier abnormality, and susceptibility to skin infection [7]. Although not always recognized by health-care professionals as being a serious medical condition, AE can have a significant negative impact on quality of life for children and their parents and care takers [8]. Children with AE may suffer from lack of sleep, irritability, daytime tiredness, emotional stress, lowered self-esteem and psychological disturbance [9]. Moreover, many cases of AE clear or improve during childhood, whereas others persist into adulthood [8]. Thus, there is a substantial need for cure and symptom relief as early as possible. However, despite the common claims for curative interventions, there is currently no known cure for AE in allopathic medicine [9].

Therefore, there is an increasing number of trials studying complementary and alternative medicine (CAM) to treat children with AE. There is a growing interest in CAM as a primary, maintenance, or simultaneous treatment for AE [10]. These studies suggest that CAM may improve health related quality of life of children. In fact, many people rely on these treatments as their primary approach to relieve their illness or at least to improve the duration and quality of symptomatic relief [10]. The most frequently used CAM modalities are herbal medicine, vitamins, Ayurveda, naturopathy, homeopathy, traditional healing [6], and probiotics [11]. However, current literature, published protocols and systematic reviews have not involved or included all kinds of CAM modalities. Moreover, we were not able to find any systematic review focusing on CAM with AE in children (< 14 years). Therefore, we conducted a comprehensive literature search involving CAM for AE in children (< 14 years) to add to current available evidence in order to inform clinical practice further.

## Methods

The protocol of the review was registered in PROSPERO (CRD42017071267) on 7th of August 2017 (Available from: http://www.crd.york.ac.uk/PROSPERO/). The content of the review followed the Preferred Reporting Items for Systematic Reviews and Meta-Analyses (PRISMA) [12].

### Eligibility criteria

#### Type of studies

Randomized controlled trials (RCTs) were included in the systematic review.

#### Type of participants

Children (< 14 years) diagnosed with AE by defined criteria or validated instruments or tools based on either the UK Working Party, Hanifin and Rajka (Hanifin 1980) or explicitly stated provider based diagnostic criteria [13] were included. Trials without clear diagnostic criteria but with detailed description of clinical features to be diagnosed as AE were also eligible for inclusion in a subgroup analysis. The limited age of < 14 years was set because of the maximum age as younger adolescents defined by WHO. No gender or ethnicity limitations were set.

#### Type of intervention

CAM modalities used alone or in combination with conventional therapies for children (< 14 years) were included. CAM terms have different concepts: If a non-mainstream practice is used together with conventional medicine, it’s considered as “complementary”. If a non-mainstream practice is used in place of conventional medicine, it is considered “alternative” [14]. Since a separate review on Traditional Chinese Medicine (TCM) for AE will be prepared due to clinical heterogeneity, we included the following CAM modalities: dietary advices/restriction, dietary supplements, probiotics, prebiotics, psychological interventions, oral evening primrose oil or borage oil, specific allergen immunotherapy, aromatherapy, bath therapy, bioresonance, chromotherapy, homeopathy, hypnotherapy and relaxation techniques in addition to some other CAM modalities that are known to be used for treating AE [10].

However, CAM is different from the new drug to estimate effectiveness, but to focus on its efficacy. So, it is sometimes difficult to to split CAM modalities up into parts to investigate effectiveness and safety of CAM modalities separately, except the placebo-controlled randomized trials [15]. Therefore, from the component level, CAM in the intervention group can be classified as above specific modalities such as probiotics, bath therapy, and so on. And from the system level, CAM can be considered as an integrated “whole system” of intervention.

#### Type of outcomes

Primary outcomes included clinical disease severity measured by one or more of the following instruments: (1) global improvement in objective AE outcomes as measured by scoring atopic dermatitis index (SCORAD); eczema area and severity index score (EASI); Nottingham eczema severity score (NESS) reported by a clinician; global improvement in subjective AE outcomes as measured by patient oriented eczema measure (POEM); itching visual analogue score (VAS); dermatology life quality index (DLQI) reported by participants or their parents. (2) Frequency of treatment discontinuation due to adverse effects. Secondary outcomes included (1) relapse rate; (2) proportion of participants with ≥50% symptoms and signs improvement in a given outcome as assessed by a clinician; (3) type, frequency, and severity of adverse events.

### Search strategy

We conducted systematic literature searches in 12 electronic databases, including 4 Chinese databases (China National Knowledge Infrastructure (CNKI), Wanfang Database, Chinese Scientific Journal Database (VIP), and SinoMed), and 8 English databases: PubMed, EMBASE via OVID, AMED (Allied and Complementary Medicine Database) via OVID, CINAHL (Cumulative Index to Nursing and Allied Health Literature) via EBSCO, PsychoInfo, CAM-QUEST, the GREAT database (the Global Resource for Eczema Trials: www.Greatdatabase.org.uk), and the Cochrane Library from their inception date until May 2018. The filters were English and Chinese language (Additional file 1). We also searched in the grey literature such as conference proceedings and dissertations in CNKI and Wanfang for unpublished trials and trial protocols. References of all included studies were hand searched for additional eligible studies.

### Study selection and data extraction

Two authors (CL Lu and SB Liang) independently examined the full text to identify the eligible trials. Four authors in pair (CL Lu, XH Liu, X Wang, and X Bai) extracted data independently from the included studies according to a predesigned data sheet. Any disagreement was resolved by discussion with a third author (JP Liu). Following items were extracted: publication year, study type, funding, inclusion/exclusion criteria, diagnostic criteria, study methodology, demographic characteristics of the participants, details of intervention and controls, outcome measures methods, adverse events, and results.

### Quality assessment

Two authors (CL Lu and XH Liu) used the risk of bias tool [16] to assess the methodological quality of the included trials. Seven items including random sequence generation, allocation concealment, blinding of participants and personnel, blinding of outcome assessment, incomplete outcome data, selective reporting and other bias such as pharmaceutical funding, were used to be judged as “low risk”, “high risk”, or “unclear risk”. Any disagreements were resolved by discussion with a third author (JP Liu).

### Data analysis

We used RevMan 5.3 software for data analysis. For continuous data, we used mean difference (MD) with 95% confidence intervals (CI), and for dichotomous data we used relative risk (RR) with 95% CI. We performed meta-analyses for trials if the study design, participants, interventions, control, and outcome measures were similar. Bulk data were synthesized quantitatively by descriptive counting. Other data not suitable for pooling analysis were synthesized qualitatively.

We used I-square (I^2^) to test the statistical heterogeneity as recommended by the Cochrane Handbook for Systematic Reviews of Interventions (Higgins 2011). We considered I^2^ statistic value greater than 50% as a suggestion that there might be substantial heterogeneity [16]. We used random effects model for data pooling with significant heterogeneity (*I*^*2*^ ≥ 50%), otherwise a fixed effect model was applied. If the data were available, we did subgroup analyses for subcategories of CAM modalities.

A sensitivity analysis was conducted to explore the influence of the type of randomized trials (parallel or cross-over randomized) and the quality of trials (high or low) if the data were available. A funnel plot was generated to explore possible publication bias if more than ten trials were included in a meta-analysis.

## Results

### Description of studies

**Fig. 1:**
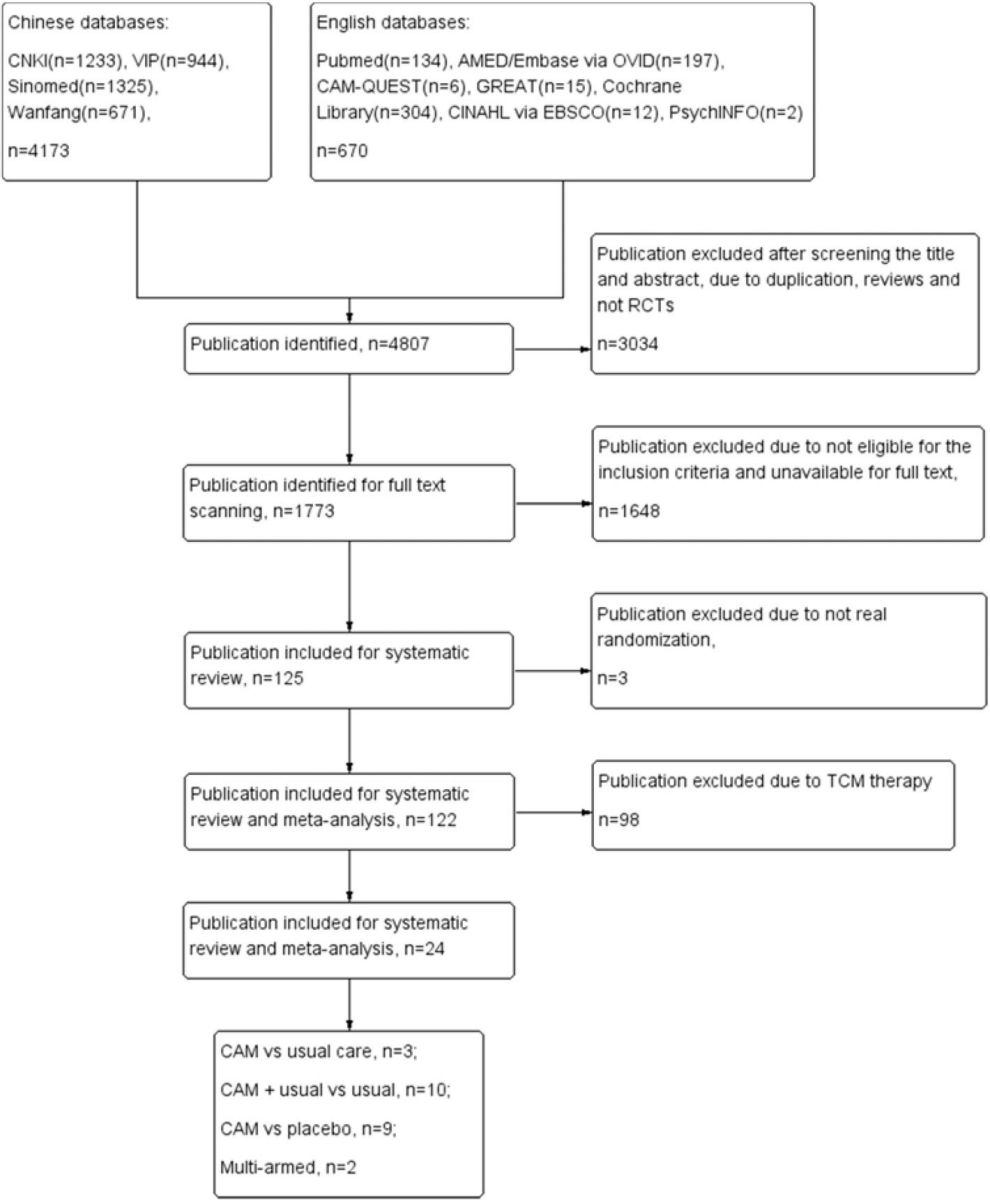
Flow diagram of study selection and different sub-groups interventions included in this review

Our searches identified 4807 citations. After reviewing the titles and abstracts, 3034 citations were excluded due to duplication, reviews, and non RCTs. After scanning the full texts to identify the participants who were over 14 years, we excluded 1648 publications. Among 125 publications that were eligible, three publications were excluded [17–19] due to inappropriate allocation of participants. We excluded 98 trials for the intervention of TCM in separate systematic review. Finally, there were 24 trials [20–43] with a total of 2233 children (< 14 years) included in this review (Fig. 1). Eleven trials [33–43] were published in English, and 13 trials [20–32] were in Chinese. We did not identify any unpublished study. Twenty-two trials [20–41] had two arms with parallel groups, one trial [42] had three arms, and one trial [43] had five arms.

### Study characteristics

The details of the 24 trials are presented in Table 1. The sample size of these studies ranged from 15 to 298 participants. The age ranged from 2 months to 13 years. We defined the conventional therapy with more than two modalities (e.g. topical and systemic anti-allergic, and immunomodulatory therapy) as “usual care” in 24 trials [9]. Every trial had more than two modalities of conventional therapy except placebo. Therefore, from the component level, CAM modalities of 22 trials [21, 23–43] in the intervention group could be classified as probiotics, diet, biofilm, and borage oil (undershirts coated with oil) (more in Table 2). Moreover, two trials [20, 22] had their main modality referring to swimming and biofilm while accompanying other modalities. So, we considered for three different comparisons in the two-arm trials from system level: CAM versus usual care [20–22], CAM plus usual care versus usual care [23–32], and CAM (probiotics) versus placebo [33–41]. For the two trials with three or more arms [42, 43], probiotics was compared with other formula of probiotics, placebo, usual care, or observation with no intervention.

**Table 1:** Characteristics of included randomized clinical trials on CAM therapies for childhood atopic eczema

Study ID	Sample size	Age	Sex M/F	Comparisons	Outcome	Follow up
CAM vs usual care, 3 studies						
Liu CH 2009 [20]	T:150 C:148	T:1-6 m, 80 cases; 6-12 m, 50 cases; 1-2 y, 20 cases C:1-6 m, 83 cases; 6-12 m, 44 cases; 1-2 y, 21 cases	T:88/62 C:86/44	Swimming therapy + Baibu (*Stemona japonica*) lotion (bath) + Tuina vs Cyproheptadine (oral) + Boric lotion (external application with cold lotion)/Hydrocortisone butyrate cream (external use)/Zinc oxide cream (external use) (7-15d)	Improvement of symptoms and signs and signs; IgG; Relapse (T:26/150, 3 m; 37/150, 6 m C:80/148, 3 m; 96/148, 6 m)	3 m, 6 m
Liu WQ 2016 [21]	T:60 C:60	T:(4.5 ± 3.8) y C:(5.3 ± 4.5) y	T:37/23 C:33/27	Fasting and rotation diet vs Pevisone paste (external use) (3 m)	Improvement of symptoms and signs; IgG; Relapse (T:4/60 C:11/60)	3 m
Wu YQ 2014 [22]	T:74 C:74	T:(6.35 ± 1.36) m C:(5.98 ± 1.23) m	T:41/33 C:31/43	Velvetfeeling lotion (external application) + Moisturizing cream (external use) + Saline water (dipping) vs Boric lotion (external application) + Vitamin E (external use) + Saline water (dipping) (7d)	Improvement of symptoms and signs; CGI-EI	NR
CAM + usual care vs usual care, 10 studies						
Chen DX 2015 [23]	T:20 C:20	T:(3.82 ± 0.7) m C:(2.35 ± 1.3) m	NR	Bifid triple viable capsules (oral) + Hydrocortisone butyrate cream (external use) vs Hydrocortisone butyrate cream (external use) (180d)	Improvement of symptoms and signs	6 m
Chen YL 2015 [24]	T:58 C:58	T:(11 ± 5) m C:(12 ± 5) m	T:31/27 C:30/28	Probiotics (oral) + Chlorphenamine maleate tablets (oral) + Vitamin B6 + Fluocinonide cream (external use) + Calcium supplement (oral) vs Chlorphenamine maleate tablets (oral) + Vitamin B6 + Fluocinonide Cream (external use) + Calcium supplement (oral) (28d)	Improvement of symptoms and signs; CGI-EI; Interleukin; Interferon; Relapse (T:9/58 C:29/58)	NR
Guo YH 2015 [25]	T:90 C:90	T:2 m-3 y C:2 m-3 y	total: 98/82	Tetralogy of viable bifidobacterium tablets (oral) + Usual care (Calamine lotion/ Zinc oxide cream/ Loratadine syrup/ Mometasone furoate cream) vs Usual care (Calamine lotion/Zinc oxide cream/Loratadine syrup/Mometasone furoate cream) (30d)	Improvement of symptoms and signs; IL-4, IL-10, IFN-γ, IgE, Th1/Th2; Relapse (T:24/90 C:62/90)	3 m
Jiang YX 2013 [26]	T:65 C:60	T:2 y C:2 y	total: 72/53	Velvetfeeling lotion (external application) + Usual care (eg. Chlorphenamine maleate tablets) vs Usual care (eg. Chlorphenamine maleate tablets) + Boric lotion (external application) (28d)	Improvement of symptoms and signs	NR
Li DY 2012 [27]	T:32 C:30	T:(7.15 ± 2.06) m C:(6.89 ± 2.54) m	T:17/15 C:16/14	Bifid triple viable capsules (oral) + Zinc oxide cream (external use) + Boric lotion (external application) vs Zinc oxide cream (external use) + Boric lotion (external application) (14d drugs for external use/28d Oral bifid-triple viable capsule)	Improvement of symptoms and signs; Relapse (T:6/32 C:20/30)	NR
Mao HX 2013 [28]	T:50 C:50	T:2 m-5 y C:2 m-5 y	T:24/26 C:28/22	Probiotics (oral) + Antihistamines (oral) + Calcium supplement (oral) + Skincare cream (external use) vs Antihistamines (oral) + Calcium supplement (oral) + Skincare cream (external use)	Improvement of symptoms and signs; Relapse (T:2/50 C:12/50)	NR
Wei MX 2010 [29]	T:38 C:36	T:(6.78 ± 2.62) m C:(7.14 ± 2.10) m	T:22/16 C:19/17	Viable *Bacillus coagulans* tablets (oral) + Boric lotion (wash-out) + Zinc oxide cream (external use) vs Boric lotion (wash-out) + Zinc oxide cream (external use) (28d)	Improvement of symptoms and signs; Relapse (T:7/38 C:24/36)	6 m
Ye CQ 2017 [30]	T:48 C:48	T:(6.9 ± 2.4) m C:(6.8 ± 2.6) m	T:27/21 C:28/20	Condensation living bacterium bacillus (oral) + Boric lotion (wash-out) + Zinc oxide cream (external use) vs Boric lotion (wash-out) + Zinc oxide cream (external use) (28d)	Improvement of symptoms and signs; Relapse (T:6/48 C:28/48)	6 m
Zhang MH 2013 [31]	T:35 C:35	T:(5 ± 3) m C:(6 ± 3) m	T:27/8 C:25/10	Bifico lriple viable (oral) + Boric lotion (wash-out) + Zinc oxide cream (external use) + Cetirizine hydrochloride drops (oral) vs Boric lotion (wash-out) + Zinc oxide cream (external use) + Cetirizine hydrochloride drops (oral) (30d)	IFN-γ; Interleukin; T-Cell; Relapse (T:6/35 C:16/58)	6 m
Zhang XN 2013 [32]	T:36 C:34	total:(7.06 ± 3.48) y	total:32/38	Velvetfeeling lotion (external application) + Butyl flufenamate ointment (external use) + Antihistamine (oral) vs Saline water (external application) + Butyl flufenamate ointment (external use) + Antihistamine (oral) (14d)	Improvement of symptoms and signs; CGI-EI	NR
CAM vs placebo, 9 studies						
D. Sistek 2006 [33]	T:30 C:29	T:3.8 y C:4.4 y	T:15/14 C:17/13	Probiotics (oral) vs Placebo (oral) (12w)	Improvement of symptoms and signs	4w
Hyeon-Jong Yang 2014 [34]	T:50 C:50	T:(58.7 ± 29.9) m C:(47.4 ± 28.1) m	T:29/21 C:24/26	Probiotics (oral) vs Placebo (oral) (6w)	Improvement of symptoms and signs; Fecal cell counts; IL-4; TNF-α	NR
Reza 2011 [35]	T:19 C:21	T:(28.68 ± 40.86) m C:(22.76 ± 34.03) m	T:11/8 C:14/7	Synbiotic (oral) vs Placebo (oral) (8w)	Improvement of symptoms and signs Mononuclear cells	NR
S Weston 2017 [36]	T:28 C:28	T:(11.5 ± 4.2) m C:(10.3 ± 3.2) m	T:14/14 C:16/12	VRI-003 PCC freeze dried powder probiotics (oral) vs Placebo (oral) (8w)	Improvement of symptoms and signs; total IgE levels; radioallergosorbent test	8w
Sergei V. Gerasimov 2010 [37]	T:48 C:48	T:(25.6 ± 7.7) m C:(24.1 ± 6.3) m	T:28/15 after withdrew C:28/19 after withdrew	Probiotics (oral) vs Placebo (oral) (8w)	Improvement of symptoms and signs; Quality of life; Total IgE; Eosinophil count	NR
Shoko 2007 [38]	T:16 C:16	T:4.44 y C:5.56 y	T:9/7 C:12/4	Borage oil (undershirts coated with oil) vs Placebo (non-coated undershirts) (2w)	Improvement of symptoms and signs; Changes of transepidermal water loss	NR
Wu YJ 2017 [39]	T:33 C:33	T:(1.5 ± 1.1) m C:(7.14 ± 2.10) m	T:25/8 C:19/14	Probiotics (Lactobacillus rhamnosus) (oral) vs Placebo (oral) (8w)	Improvement of symptoms and signs; Quality of Life (Infant Dermatitis Quality of Life Questionnaires and Dermatitis Family Impact Questionnaires);	NR
Yavuz 2012 [40]	T:20 C:20	total:1-13 y	total: 23/17	Probiotic bacteria (oral) vs Placebo (oral) (8w)	Improvement of symptoms and signs; cytokine analyse/IgE/Eosinophil cationic protein	10w
Youngshin 2012 [41]	T:58 C:60	T:(4.6 ± 3.3) y C:(5.1 ± 3.3) y	T:34/24 C:35/25	Probiotics (*L. plantarum* CJLP133) (oral) vs Placebo (oral) (12w)	Improvement of symptoms and signs; total IgE levels/specific IgE	2w
Multi-armed trials, 2 studies						
Pasi E. Kankaanpa 2002 [42]	T1:5 T2:5 C1:5	T1:(4.5 ± 2) m T2:(5.7 ± 2.2) m C1:(5.6 ± 2.1) m	T1:2/3 T2:3/2 C1:2/3	Probiotics (Lactobacillus GG) (oral) vs Probiotics (Bifidobacterium Bb12) (oral) vs placebo (oral) (①4.4 ± 1.7 m ②7.3 ± 0.7 m ③5.7 ± 2.0 m)	Fatty acid analysis	≥36 m follow until panticipants at the age of 3 years
C. Gore 2011 [43]	T1:45 T2:45 C1:47 C2:22 C3:49	T1:19 [16-23] w T2:20.5 [17-23] w C1:20 [16-23] w C2:15 [13-19.5] w C3:19 [15-21.5] w	T1:28/17 T2:24/21 C1:28/19 C2:16/6 C3:25/24	Probiotics (Lactobacillus paracasei) (oral) vs Probiotics (Bifidobacterium) (oral) vs Placebo (oral) vs Exclusively breastfed vs Standard formula-fed (12w)	Improvement of symptoms and signs; Quality of life; Stool; GI-Permeability; Specific serum IgE; Urinary EPX	NR

*T* treatment group, *C* control group, *y* year, *m* month, *d* day, *w* week, *NR* not report

**Table 2:** Description and trials numbers of different CAM therapies

Therapy	Administration	Dosage	Frequency	Detail	Formula	Trials numbers	%(/22)
Probiotics	oral	depend on formula	2–3/d	Oral probiotics preparation	The probiotic formulation was a mixture of Lactobacillus acidophilus DDS-1 and Bifidobacterium lactis UABLA-12 with fructo-oligosaccharide in a rice maltodextrin powder.	18	72%
Diet	oral	None	3 m	To avoid to severe and moderate intolerant food, and take mild intolerant food per 6 days	None	2	8%
Biofilm	external application	appropriate amount	2–3/d	The main ingredient is chitosan with low polymer (OPC). External application help form a protective film on the skin.	Velvetfeeling	3	12%
Borage oil	undershirts coated withoil	498 mg of GLA per 100 g of cotton)	2 w	Borage oil was chemically bonded to the cotton fibers of the undershirts that made of pure organic cotton. The borage oil was gradually released from the cotton fibers and absorbed into the skin. The undershirts were designed such that the sutures did not touch and stimulate the skin directly.	Borage oil	1	4%
Swimming	Passively by nurse; Autonomous swimming	None	1/d	Trained nurse helps baby to stretch the limb as passively swimming or baby swim autonomously.	None	1	4%

*m* month, *d* day, *w* week

### Risk of bias of included trials

**Fig. 2:**
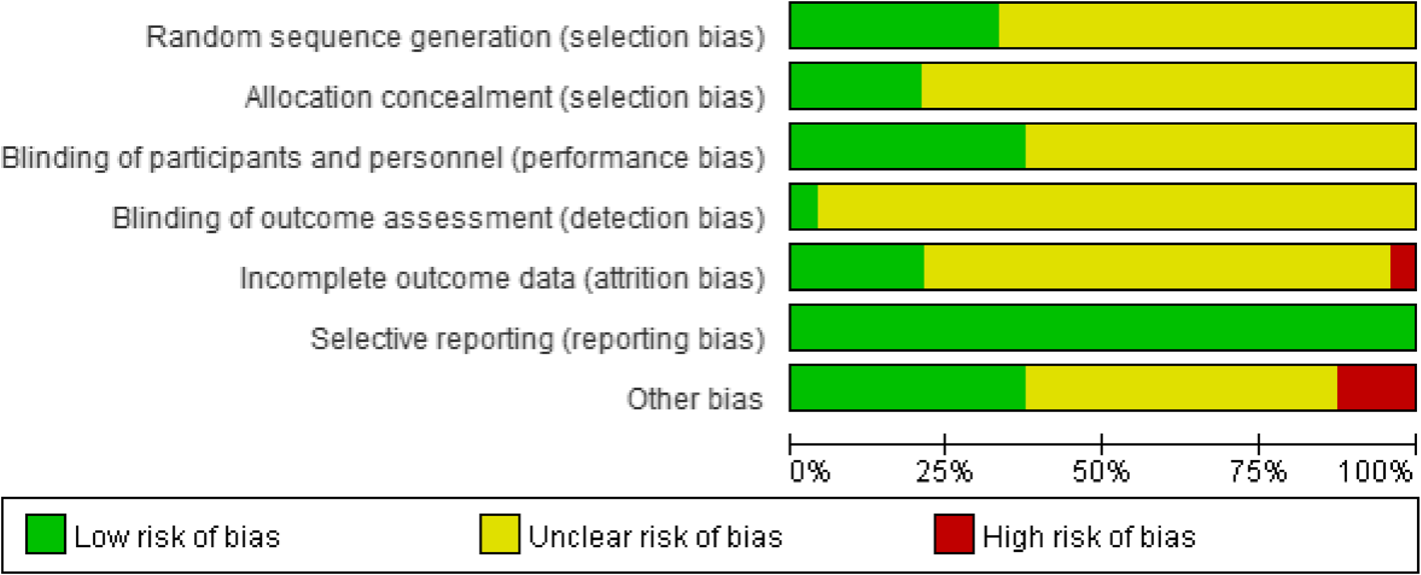
Risk of bias graph

The trials only reporting that the study was “randomized” were defined as “unclear” risk of bias, while the trials describing a specific method of randomized sequences generation, allocation concealment, and blinding as “low” risk of bias. Other bias accessed the funding scheme. Trials supported by non-commercial funding were defined as “low” risk of bias, trials funded by pharmaceutical companies were classified as “high” risk of bias, no information as “unclear” risk of bias. Eight trials [22, 24, 29, 31, 33, 34, 37, 41] reported the random allocation by random number table or computer generated-list. Only five trials [33, 34, 37, 38, 40, 41] reported the allocation concealment by using computer-generated random numbers or randomization software, which can conceal the allocation automatically. Ten trials [33, 34, 36–43] reported double blinding. Eight trials [33–37, 39, 41, 43] reported the drop out in both intervention group and control group, and only four trials [33, 39, 41, 43] used intention-to-treat (ITT) to analyze for all outcome [33, 39, 41] and primary outcome [43], and the other four trials [34–37] analyzed data by per-protocol (PP) and reported the data of available participants. Besides, two trials [39, 41] analyzed by both ITT and PP. We considered one trial [34] as high risk of incomplete data for loss to follow up without ITT analysis because of 13 withdrawals in intervention group and 16 withdrawals in control group among the 100 participants in the trial. Four trials [33, 39, 41, 43] were considered as low risk of bias and the others as unclear. Eleven trials [21, 25, 29, 33, 35–37, 40–43] mentioned non-commercial funding. Three trials [33, 39, 43] reported that probiotic manufacturers produced the drugs used. We considered these three trials [33, 39, 43] as high risk of bias for conflict of interest (Fig. 2). Sample size calculation was reported in five trials [33, 35, 37, 41, 43] according to disease prevalence [33], symptoms and signs reduction in treatment group by 30% [34, 41], 34% [37] and in placebo group by 15% [34], 17% [37], 10% [41], and symptoms and signs scale of SCORAD for a standard deviation increments of 7.65 [43]. The other trials did not report any detail of sample size calculation. We considered the other bias of power calculation as unclear.

### Effects of interventions

The 24 trials tested five CAM interventions: combination of probiotics (72%, *n* = 18) [23–25, 27–43], biofilm (12%, *n* = 3) [22, 26, 32], diet (8%, *n* = 2) [21, 43], swimming (4%, *n* = 1) [20], and undershirts coated with borage oil (4%, *n* = 1) [38] (Table 2).

Twenty-two two-arms trials [20–41] involved CAM modalities, including probiotics, swimming, diet, borage oil, and biofilm. Three different comparisons were summarized: CAM versus placebo, CAM versus usual care, and CAM plus usual care versus usual care alone. In addition to this, two multi-arm trials [42, 43] used different probiotic formulae in different groups to compare with placebo, observation with no intervention or diet. Table 3 showed the detailed results of the effect estimation.

**Table 3:** Summary of findings of CAM for childhood atopic eczema in randomized controlled trials

Study ID	Sample size	Main intervention	Estimate effect [95% CI]	Outcome	*P*
CAM vs usual care					
Liu CH 2009 [20]	T:150 C:148	Swimming therapy	RR 1.01 [0.92, 1.11] RR 1.01 [0.96, 1.07] RR 0.38 [0.28, 0.52]	Clinical effectiveness rate 50% Improvement of symptoms and signs Relapse rate	*P* = 0.8318 *P* = 0.5671 *P* < 0.00001
Liu WQ 2016 [21]	T:60 C:60	Fasting and Rotation diet	RR 1.57 [1.04, 2.38] RR 1.32 [1.09, 1.60] RR 0.36 [0.12, 1.08]	Clinical effectiveness 50% Improvement of symptoms and signs; Relapse rate	*P* = 0.0323 *P* = 0.0049 *P* = 0.0681
Wu YQ 2014 [22]	T:74 C:74	Velvetfeeling lotion (external application)	RR 1.92 [1.36, 2.72] RR 1.33 [1.10, 1.61]	Clinical effectiveness rate 50% Improvement of symptoms and signs	*P* = 0.0002 *P* = 0.0031
CAM + usual care vs usual care					
Chen DX 2015 [23]	T:20 C:20	Bifid Triple Viable capsules (oral)	RR 1.63 [0.87, 3.04] RR 1.73 [1.15, 2.60]	Clinical effectiveness rate 50% Improvement of symptoms and signs	*P* = 0.1283 *P* = 0.0088
Chen YL 2015 [24]	T:58 C:58	Probiotics (oral)	RR 1.21 [0.87, 1.68] RR 1.19 [0.99, 1.42] RR 0.31 [0.16, 0.60]	Clinical effectiveness rate 50% Improvement of symptoms and signs Relapse rate	*P* = 0.2659 *P* = 0.0624 *P* = 0.0004
Guo YH 2015 [25]	T:90 C:90	Tetralogy of viable bifidobacterium tablets (oral)	RR 1.37 [1.01, 1.85] RR 1.27 [1.04, 1.55] RR 0.39 [0.27, 0.56]	Clinical effectiveness rate 50% Improvement of symptoms and signs Relapse rate	*P* = 0.0400 *P* = 0.0172 *P* < 0.00001
Jiang YX 2013 [26]	T:65 C:60	Velvetfeeling lotion (external application)	RR 1.85 [0.90, 3.79] RR 1.91 [1.46, 2.50]	Clinical effectiveness rate 50% Improvement of symptoms and signs	*P* = 0.0947 *P* < 0.00001
Li DY 2012 [27]	T:32 C:30	Bifid Triple Viable capsules (oral)	RR 1.36 [1.03, 1.79] RR 1.32 [1.06, 1.65] RR 0.28 [0.13, 0.60]	Clinical effectiveness rate 50% Improvement of symptoms and signs; Relapse rate	*P* = 0.0295 P = 0.0151 *P* = 0.0011
Mao HX 2013 [28]	T:50 C:50	Probiotics (oral)	RR 0.17 [0.04, 0.71]	Relapse rate	*P* = 0.0151
Wei MX 2010 [29]	T:38 C:36	Viable Bacillus Coagulans tablets (oral)	RR 1.33 [1.05, 1.68] RR 1.30 [1.07, 1.58] RR 0.28 [0.14, 0.56]	Clinical effectiveness rate 50% Improvement of symptoms and signs; Relapse rate	*P* = 0.0189 *P* = 0.0089 *P* = 0.0004
Ye CQ 2017 [30]	T:48 C:48	Condensation living bacterium bacillus (oral)	RR 1.57 [1.21, 2.02] RR 1.31 [1.10, 1.55] RR 0.21 [0.10, 0.47]	Clinical effectiveness rate 50% Improvement of symptoms and signs; Relapse rate	*P* = 0.0005 *P* = 0.0019 *P* = 0.0001
Zhang MH 2013 [31]	T:35 C:35	Bifico Lriple Viable (oral)	RR 1.15 [0.91, 1.46] RR 0.38 [0.17, 0.85]	50% Improvement of symptoms and signs; Relapse rate	*P* = 0.2371 *P* = 0.0180
Zhang XN 2013 [32]	T:36 C:34	Velvetfeeling lotion (external application)	RR 6.61 [1.62, 26.96] RR 1.25 [1.00, 1.56]	Clinical effectiveness rate 50% Improvement of symptoms and signs	*P* = 0.0084 *P* = 0.0542
CAM vs placebo					
Reza 2011 [35]	T:19 C:21	Synbiotic (oral)	MD 19.10 [7.60, 30.60]	Clinical effectiveness scores	*P* = 0.0017
Sergei V. Gerasimov 2010 [37]	T:48 C:48	Probiotics (oral)	MD 6.40 [2.71, 10.09]	Clinical effectiveness scores	*P* = 0.0009
Wu YJ 2017 [39]	T:33 C:33	Probiotics (Lactobacillus rhamnosus) (oral)	MD 10.85 [3.82, 17.88]	Clinical effectiveness scores	*P* = 0.0035
Yavuz 2012 [40]	T:20 C:20	Probiotic (oral)	MD 10.20 [7.45, 12.95]	Clinical effectiveness scores	*P* < 0.00001
Youngshin 2012 [41]	T:58 C:60	Probiotics (L. plantarum CJLP133) (oral)	MD 7.30 [2.63, 11.97]	Clinical effectiveness scores	*P* = 0.0029

*CAM* complementary and alternative medicine, *RR* risk ratio, *MD* mean difference, *CI* confidence interval

#### Global improvement (symptoms and signs improvement ≥95%, such as itching, skin lesions, swelling, and papula)

Global improvement was better for CAM (probiotics) compared with placebo in five trials [35, 37, 39–41] including 323 participants (MD 9.01, 7.12–10.90; *I*^*2*^ = 37%). Three trials [20–22] with 566 participants showed no difference between CAM alone (swimming, diet, or biofilm) and usual care (RR 1.43, 0.82–2.48; *I*^*2*^ = 91%). Eight trials [23–27, 29, 30, 32] involving 763 participants showed better effect from CAM plus usual care (RR 1.47, 1.30–1.68; *I*^*2*^ = 11%) compared with usual care.

Apart from statistical heterogeneity, the interventions in three trials [20–22] were totally different, including swimming [20], diet [21], biofilm [22], and one trial [20] investigated swimming in combination with Chinese herbal medicine lotion and tuina (Chinese massage for children). We conducted a qualitative description on these three trials. One trial [20] tested swimming, Chinese herbal medicine lotion and tuina compared to usual care. The intervention group showed more symptom reduction than the control group, however, not at a significant level. One trial [21] compared fasting and rotation diet with Pevisone paste, and reported beneficial effects of diet on symptom improvement. Another trial [22] showed statistically significant effects of Velvetfeeling Lotion (biofilm) on symptom improvement when compared with usual care.

#### Relapse rate

CAM showed lower relapse rate compared to usual care (RR 0.38, 0.28–0.51; *n* = 2, 418 participants) [20, 21]. CAM plus usual care showed lower relapse rate compared to usual care (RR 0.31, 0.24–0.40; *n* = 7, 698 participants) [24, 25, 27–31]. Nine trials [33–41] with 622 participants compared probiotics with placebo, but did not report the relapse, and rest of two trials [22, 23] with 188 participants did not report the relapse either.

#### ≥50% improvement of symptoms and signs (such as itching, skin lesions, swelling, and papula)

Three trials [20–22] with 566 participants showed no difference between CAM alone (RR 1.20, 0.90–1.60; *I*^*2*^ = 92%) and usual care. Nine trials [23–27, 29–32] with 833 participants showed improvement from CAM (RR 1.34, 1.25–1.45; *I*^*2*^ = 35%) in addition to usual care compared with usual care alone. Nine trials [33–41] with 622 participants compared probiotics with placebo reported as continuous data resulting in unavailable outcome for ≥50% improvement of symptoms.

Apart from statistical heterogeneity, three trials had clinical heterogeneity for different CAM modalities [20–22]. One trial [20] showed that swimming had more children with improvement of symptoms and signs of ≥50% than the control group, but not at a significant level. One trial [21] showed significant effects of diet. Another trial [22] reported the positive effect of biofilm compared with usual care.

#### Adverse events

Only 12 trials (50%) [20, 22, 24, 25, 29, 30, 32, 35–37, 39, 43] reported the outcome of adverse events. Among these, four trials [27, 29, 35, 36] reported no occurrence of adverse events in either groups, and one trial [39] reported no relation between adverse events and the tested product without any details about adverse events, while seven trials [20, 22, 24, 30, 32, 37, 43] reported that children (< 14 years) with adverse events gradually adapted to treatments without extra treatment or that the adverse events was not related to the medications under investigation (Table 4). No severe adverse event such as death or hospitalization were reported. The reported adverse events included crying, irritability, and worsening of skin lesions (reddening) (Table 4).

**Table 4:** Adverse events of CAM for childhood atopic eczema in randomized controlled trials

Study ID	Total sample num	Sample num in intervention group	Sample num in control group	Adverse events cases	Intervention group	Control group	Treatment for adverse event
C. Gore 2011 [43]	208	137	71	T: 42/137 C: NR	green loose stools; increased vomiting; feed-refusal; colic	NR	24/137 (17.5%) participants stopped the study formula.
Chen YL 2015 [24]	116	58	58	T: 1/58 C: 2/58	1 for dizzy.	1 for dizzy; 1 for drowsy.	NR
Liu CH 2009 [20]	298	150	148	T: 0/150 C: 57/148	None	21 for facial flushing; 18 for dry skin; 8 for partial facial skin thinning; 10 for facial skin with mild pigmentation; Unclear for other hormonal dermatitis symptoms.	NR
Sergei V. Gerasimov 2010 [37]	96	48	48	T: 26/48 C: 24/48	11 for upper respiratory tract infections; 4 for lower respiratory tract infections; 7 for herpetic stomatitis; 3 for diarrhea; 6 for constipation; 5 for abdominal colic; 2 for burn and croup with severe adverse events.	10 for upper respiratory tract infections; 5 for lower respiratory tract infections; 5 for herpetic stomatitis; 2 for diarrhea; 6 for constipation; 4 for abdominal colic; 3for head injury, food poisoning with severe adverse events.	None was related to the medications under investigation.
Wu YQ 2014 [22]	148	74	74	T: 2/74 C: 5/74	2 for crying and mildly red skin lesions on the 2nd day.	5 for crying, irritability, and red skin lesions.	Without any treatment, to ease soon and symptoms disappearance.
Ye CQ 2017 [30]	96	48	48	T:3/48 C:14/48	1 for diarrhea; 2 for constipation.	6 for diarrhea; 8 for constipation.	NR
Zhang XN 2013 [32]	70	36	34	T: 1/36 C: 2/34	1 for mild skin irritation after using Velvetfeeling Lotion	2 forskin lesions reddening after using Butyl Flufenamate Ointment.	Without any treatment, to ease soon and symptoms disappearance.

*T* treatment group, *C* control group, *NR* not report

### Additional analysis

Since the fact that each comparison did not include more than 10 trials, we were not able to conduct meaningful funnel plot analysis in order to identify the publication bias. Due to the same quality and the type of randomized trials, we could not conduct sensitivity analysis in this aspect. Besides, significant heterogeneity in two outcomes with two comparisons was more than 50% (*I*^*2*^ ≥ 50%), so we conducted a subgroup meta-analysis or a meaningful sensitivity analysis.

The global improvement (≥95% improvement) and ≥ 50% improvement of symptoms and signs in probiotics compared with usual care in three trials with 566 participants [20–22] showed I^2^ as 91 and 92%. The interventions were very heterogeneous in the trials including swimming [20], diet [21], and biofilm [22]. One trial [20] investigated not only swimming but also Chinese herbal medicine lotion and tuina. We conducted a sensitivity analysis, which showed improvement from CAM both for global improvement ≥95% improvement) (RR 1.77, 1.36–2.31; *n* = 2) and ≥ 50% improvement of symptoms and signs (RR 1.33, 1.16–1.52; *n* = 2).

## Discussion

### Summary of findings

This review identified 24 RCTs involving 2233 children (< 14 years) with AE. The findings suggest that some of the CAM modalities used alone or in combination with usual care may relieve the symptoms and signs of AE with ≥95% and ≥ 50% improvement, such as itchiness, skin lesions, swelling, and papula, in addition to reduce relapse of eczema. Moreover, some CAM modalities (such as probiotics) showed significant effect compared with placebo. The evaluated modalities appear to be safe and tolerated for lower relapse rate in CAM modality group. In spite of unclear pathogenesis of AE, CAM modalities may reduce symptoms and signs, and relapse of AE compared to conventional therapies.

The majority of trials had unclear risk of bias in many domains such as allocation concealment, blinding, missing data, and sample size calculation. Due to the unclear risk of bias of included trials, we could not come to firm conclusions from the evidence of the included trials in this review.

### Comparison with previous studies

By searching the Cochrane Library with “AE”, “AD”, and “CAM”, there are 19 Cochrane reviews and 11 other reviews published, and after scanning, 9 reviews [13, 44–51] with CAM related to children (< 14 years) for treating or preventing. These reviews or protocols included both children and adults, and even pregnant women to prevent, cure and explore the pathogenesis of AE. We found only one protocol [44] similar to our review, but was withdrawn as the author gave up the title “Complementary and alternative medicine treatments for AE”. In addition, the previous studies did not exclude the allergic diseases (such as asthma, and intestinal diseases) to focus on AE. Our review included children (< 14 years) suffering only from AE. Moreover, most reviews investigated the specific treatment like probiotics, based on pathological mechanisms but ignoring the complex and unclear pathogenesis of AE. The findings of our review are based on the symptom relief of AE and we included more comprehensive trials involving CAM for children (< 14 years).

### Strengths and limitations

Although great effort was made to retrieve all trials, we still cannot confirm that we were able to cover all the evidence due to non identified unpublished data. Besides, selecting and extracting data may also lead to some bias. We included only children under the age of 14 as this is the maximum age as younger adolescents defined by WHO, which may exclude some studies due to unavailable data for their participants over the age of 14 years. In addition, due to the various treatment for AE with an integrated “whole system” of care approach, we considered the control group with usual care as the system effect. The intervention group with CAM for a specific modality as the component efficacy, which cannot be used to document or disprove the effectiveness of a “whole system” treatment approach [15]. Additionally, in terms of the statistical heterogeneity and the variability in the CAM modalities, we were not able to conduct a subgroup meta-analysis, meaningful sensitivity analysis and funnel plot analysis. These factors limit the conclusiveness and robustness of this systematic review.

### Implications for research

In fact of the limitations of this review, future trials should be designed as multi-center, double-blind placebo controlled trials with sufficient power, and reported according to the CONSORT (Consolidated Standards for Reporting Trials) Statement [52]. In addition, trials should record the relapse with sufficient length of follow-up. Besides, it is important to provide the definite safe treatments to the patients. Thus, adverse events in each group should be reported respectively so that we could retrospect the reason of different modalities and be easy to estimate the safety of CAM modalities.

## Conclusion

Based on evidence from this systematic review we found some promising effect of CAM modalities on reducing symptoms and signs, and relapse of AE. However, it is still premature to recommend the therapy in clinical practice due to the limited number of trials and general low methodological quality of the included trials. Further rigorously double-blind, placebo-controlled trials are warranted to confirm efficacy of the CAM modalities for AE.

## Additional file

Additional file 1: Searching strategy for electronic databases. (DOCX 20 kb)

## Abbreviations

< 14 years: Under 14 years old
AD: Atopic dermatitis
AE: Atopic eczema
CAM: Complementary and alternative medicine
CHM: Chinese herbal medicine
CI: Confidence interval
CNKI: China National Knowledge Infrastructure
DLQI: Dermatology life quality index
EASI: Eczema area and severity index score
I^2^: I-square
ITT: Intention-to-treat
MD: Mean difference
NESS: Nottingham eczema severity score
POEM: Patient oriented eczema measure
PP: Per-protocol
RCTs: Randomized clinical trials
RR: Relative risk
SCORAD: Scoring atopic dermatitis index
SMD: Standardized mean difference
VAS: Itching visual analogue score
VIP: Chinese Scientific Journal Database

## References

[1] Helen N, Alan M, Williams HC (2011). Mapping randomized controlled trials of treatments for eczema-The GREAT database (The Global Resource of Eczema Trials: a collection of key data on randomized controlled trials of treatments for eczema from 2000 to 2010). BMC Dermatol.

[2] Manjra AI, Du PP, Weiss R (2005). Childhood atopic eczema consensus document. S Afr Med J.

[3] Nutten S (2015). Atopic dermatitis: global epidemiology and risk factors. Ann Nutr Metab.

[4] Shaw TE, Currie GP, Koudelka CW (2011). Eczema prevalence in the United States: data from the 2003 national survey of children’s health. J Investig Dermatol.

[5] Genuneit J, Seibold AM, Apfelbacher CJ (2017). Overview of systematic reviews in allergy epidemiology. Allergy.

[6] Silverberg JI, Lee-Wong M, Silverberg NB (2014). Complementary and alternative medicines and childhood eczema: a US population-based study. Dermatitis.

[7] McAleer MA, Flohr C, Irvine AD (2012). Management of difficult and severe eczema in childhood. BMJ.

[8] NICE (2016). Atopic eczema in under 12s: diagnosis and management.

[9] Leung TN, Hon KL (2015). Eczema therapeutics in children: what do the clinical trials say?. Hong Kong Med J.

[10] Jadotte YT, Santer M, Vakirlis E, et al. Complementary and alternative medicine treatments for atopic eczema (Protocol). Cochrane Database Syst Rev. 2014; 10.1002/14651858.CD010938.pub2.

[11] Fuchs-Tarlovsky V, Marquez-Barba MF, Sriram K (2016). Probiotics in dermatologic practice. Nutrition.

[12] Moher D, Liberati A, Tetzlaff J (2009). Preferred reporting items for systematic reviews and meta-analyses: the PRISMA statement. PLoS Med.

[13] Gu S, Yang AWH, Xue CCL, et al. Chinese herbal medicine for atopic eczema. Cochrane Database Syst Rev. 2013; 10.1002/14651858.CD008642.pub2. 10.1002/14651858.CD008642.pub2 PMC10639001 24018636

[14] National Center for Complementary and Alternative Medicine (NCCAM). Complementary, alternative, or integrative health: what’s in a name? http://nccam.nih.gov/health/whatiscam. Accessed 7 June 2017.

[15] Fønnebø V, Grimsgaard S, Walach H (2007). Researching complementary and alternative treatments-the gatekeepers are not at home. BMC Med Res Methodol.

[16] Higgins JPT, Green S. Cochrane handbook for systematic reviews of interventions version 5.1.0.2011. https://training.cochrane.org/handbook. Accessed 7 June 2017.

[17] Wang LH, Zhou LB (2002). Clinical observation on 648 cases of infantile allergic eczema treated by “heat-clarifying and dampness-removing mixture”. J Shanghai Univ Tradit Chin Med.

[18] Zhang YZ (2013). Clinical observation on 1840 cases of infantile allergic eczema treated by qibaixiaoruan ointment. Chin Community Doct.

[19] Cheng XM (2005). Observation on the curative effect of Yinyanjing on infantile eczema. Pract Prev Med.

[20] Liu CH, Fu R, Wan H (2009). The clinical study of swimming therapy, Tuina and Baibu Heji lotion to treat infantile eczema. Hubei J Tradit Chin Med.

[21] Liu WQ, Luo DJ, Teng LZ (2016). Clinical effect observation of diet taboos combined with alternative therapy in childhood eczema with food intolerance. Med Innov China.

[22] Wu YQ, Zhao WQ, Pan JS (2014). Assessment of therapeutic effect of medical skin healing biological film combined with moisturizer on infantile acute eczema. China J Lepr Skin Dis.

[23] Chen DX (2015). Clinical effect of oral probiotics and butyric acid hydrocortisone cream in the treatment of infantile eczema. Chin Foreign Med Res.

[24] Chen YL (2015). The clinical efficacy and safety of probiotics to treat infantile eczema. China J Pharm Econ.

[25] Guo YH, Mou YD, Wang HS (2015). Clinical effect of microecologics as an adjuvant therapy on infants’ eczema. J Dalian Med Univ.

[26] Jiang YX (2013). Velvetfeeling lotion to treat 125 cases of infantile eczema. Health Horiz.

[27] Li DY (2012). The efficacy observation of bifid-triple viable capsule to treat infantile eczema. Public Med Forum Mag.

[28] Mao HX, Hu XH (2013). Clinical observation of supplement the intestinal probiotics as an adjuvant therapy to treat 50 cases of infant eczema. J Aerospace Med.

[29] Wei MX, Yan R, Luo HB (2010). The clinical observation of bacillus coagulans tablets to treat 36 cases of infantile eczema. Chin J Pract Pediatr.

[30] Ye CQ (2017). Observation of curative effect on 48 cases of infant eczema treated by coagulative bacillus granulosa bioactive tablets. China Prac Med.

[31] Zhang MH (2013). The study of adhibition and the influence of intestinal flora, immunologic function and cytokines of probiotics to treat infantile eczema. Chin J Clin Ration Drug Use.

[32] Zhang XN (2013). Observation of efficacy and safety of the velvetfeeling combined with butyl flufenamate ointment in the treatment of child eczema. China Pract Med.

[33] Sistek D, Kelly R, Wickens K (2006). Is the effect of probiotics on atopic dermatitis confined to food sensitized children?. J Brit Soc Allergy Clin Immunol.

[34] Yang HJ, Min TK, Lee HW (2014). Efficacy of probiotic therapy on atopic dermatitis in children: a randomized, double-blind, placebo-controlled trial. Allergy, Asthma Immunol Res.

[35] Reza F, Hamid A, Farahzad J (2011). Effect of a new synbiotic mixture on atopic dermatitis in children: a randomized-controlled trial. Iran J Pediatr.

[36] Weston S, Halbert A, Richmond P (2005). Effects of probiotics on atopic dermatitis: a randomised controlled trial. Arch Dis Child.

[37] Gerasimov SV, Vasjuta VV, Myhovych OO, Bondarchuk LI (2010). Probiotic supplement reduces atopic dermatitis in preschool children: a randomized, double-blind, placebo-controlled, clinical trial. Am J Clin Dermatol.

[38] Kanehara S, Ohtani T, Uede K (2007). Clinical effects of undershirts coated with borage oil on children with atopic dermatitis: a double-blind, placebo-controlled clinical trial. J Dermatol.

[39] Wu YJ, Wu WF, Hung C-W (2017). Evaluation of efficacy and safety of lactobacillus rhamnosus in children aged 4-48 months with atopic dermatitis: an 8-week, double-blind, randomized, placebo-controlled study. J Microbiol Immunol Infect.

[40] Yeşilova Y, Ömer C, Akdeniz N (2012). Effect of probiotics on the treatment of children with atopic dermatitis. Ann Dermatol.

[41] Han Y, Kim B, Ban J (2012). A randomized trial of lactobacillus plantarum CJLP133 for the treatment of atopic dermatitis. Pediatr Allergy Immunol.

[42] Kankaanpää PE, Yang B, Kallio HP (2002). Influence of probiotic supplemented infant formula on composition of plasma lipids in atopic infants. J Nutr Biochem.

[43] Gore C, Custovic A, Tannock GW (2012). Treatment and secondary prevention effects of the probiotics Lactobacillus paracasei or Bifidobacterium lactis on early infant eczema: randomized controlled trial with follow-up until age 3 years. Clin Exp Allergy.

[44] Jadotte YT, Santer M, Vakirlis E, et al. Complementary and alternative medicine treatments for atopic eczema (Protocol). Cochrane Database Syst Rev. 2017; 10.1002/14651858.CD010938.pub2.

[45] Küster D, Spuls PI, Flohr C, et al. Effects of systemic immunosuppressive therapies for moderate-to-severe eczema in children and adults (Protocol). Cochrane Database Syst Rev. 2015; 10.1002/14651858.CD011939.

[46] Kramer MS, Kakuma R. Maternal dietary antigen avoidance during pregnancy or lactation, or both, for preventing or treating atopic disease in the child. Cochrane Database Syst Rev. 2012; 10.1002/14651858.CD000133.pub3. 10.1002/14651858.CD000133.pub3 PMC7045459 22972039

[47] Osborn DA, Sinn JKH. Probiotics in infants for prevention of allergic disease and food hypersensitivity. Cochrane Database Syst Rev. 2007; 10.1002/14651858.CD006475.pub2. 10.1002/14651858.CD006475.pub2 17943912

[48] Kramer MS. Maternal antigen avoidance during lactation for preventing atopic eczema in infants. Cochrane Database Syst Rev. 1996; 10.1002/14651858.CD000131. 10.1002/14651858.CD000131 PMC10866116 10796148

[49] Bath-Hextall FJ, Delamere FM, Williams HC. Dietary exclusions for established atopic eczema. Cochrane Database Syst Rev. 2008; 10.1002/14651858.CD005203.pub2. 10.1002/14651858.CD005203.pub2 PMC6885041 18254073

[50] Bamford JTM, Ray S, Musekiwa A, et al. Oral evening primrose oil and borage oil for eczema. Cochrane Database Syst Rev. 2013; 10.1002/14651858.CD004416.pub2. 10.1002/14651858.CD004416.pub2 PMC8105655 23633319

[51] Ersser SJ, Cowdell F, Latter S, et al. Psychological and educational interventions for atopic eczema in children. Cochrane Database Syst Rev. 2014; 10.1002/14651858.CD004054.pub3. 10.1002/14651858.CD004054.pub3 PMC6457897 24399641

[52] CONSORT Statement 2001-Checklist: items to include when reporting arandomized trial. 2001. http://www.consort-statement.org. Accessed 7 June 2017.

