# Correction to: Complementary and alternative medicine for treatment of atopic eczema in children under 14 years old: a systematic review and meta-analysis of randomized controlled trials

**DOI:** 10.1186/s12906-018-2421-4

**Published:** 2019-01-14

**Authors:** Chun-li Lu, Xue-han Liu, Trine Stub, Agnete E. Kristoffersen, Shi-bing Liang, Xiao Wang, Xue Bai, Arne Johan Norheim, Frauke Musial, Terje Alræk, Vinjar Fonnebo, Jian-ping Liu

**Affiliations:** 1 Centre for Evidence-Based Chinese Medicine, Beijing University of Chinese Medicine, 100, Beijing, ,029 China; 2 Department of Community Medicine, The National Research Center in Complementary and Alternative Medicine (NAFKAM), Faculty of Health Science, UiT, The Arctic University of Norway, 9037 Tromsø, Norway; 3 School of Basic Medicine, Shanxi University of Chinese Medicine, Taiyuan, 030000 China

**Correction to: Lu et al. BMC Complementary and Alternative Medicine (2018) 18:260**

**DOI: 10.1186/s12906-018-2306-6**

Following publication of the original article [1], the author reported their family name was misspelled. The details are as follows:

Incorrect name in the original article: Terje Araek.

Correct name: Terje Alræk.
