# Supplementary material for: Complementary and alternative medicine for treatment of atopic eczema in children under 14 years old: a systematic review and meta-analysis of randomized controlled trials
Source: BMC Complement Altern Med. 2018 Sep 26;18:260. doi: 10.1186/s12906-018-2306-6 (PMC6158902; doi:10.1186/s12906-018-2306-6)
Supplement: Supplementary file 1 — Searching strategy for electronic databases. (DOCX 20 kb) [file 12906_2018_2306_MOESM1_ESM.docx]

**Additional file 1 Searching strategy for electronic databases**

| **databases** | **date** |
| --- | --- |
| PubMed | 20/05/2018 |
| #1. complementary medicine[Title/Abstract] OR alternative medicine[Title/Abstract] OR Chinese medicine[Title/Abstract] OR herbal medicine[Title/Abstract] OR acupuncture[Title/Abstract] OR massage[Title/Abstract] OR aromatherapy[Title/Abstract] OR bath therapy[Title/Abstract] OR specific allergen immunotherap[Title/Abstract] OR traditional healing[Title/Abstract] OR naturopathy[Title/Abstract] OR Ayurveda[Title/Abstract] OR vitamins[Title/Abstract] OR borage oil[Title/Abstract] OR oral evening primrose oil[Title/Abstract] OR meditation[Title/Abstract] OR mindfulness[Title/Abstract] OR psychological interventions[Title/Abstract] OR prebiotics[Title/Abstract] OR diet[Title/Abstract] OR dietary supplements[Title/Abstract] OR probiotics[Title/Abstract] OR traditional medicine[Title/Abstract]  #2. atopic eczema[Title/Abstract] OR atopic dermatitis[Title/Abstract]  #3. randomized controlled trial[Title/Abstract] OR clinical trial[Title/Abstract] OR blinding[Title/Abstract] OR placebo[Title/Abstract] OR random[Title/Abstract]  #4. English[Language]  #5. #1 AND #2 AND # 3 AND #4 | |
| The Cochrane library | 20/05/2018 |
| #1. complementary medicine[Title/Abstract/Keywords] OR alternative medicine[Title/Abstract/Keywords] OR Chinese medicine[Title/Abstract/Keywords] OR herbal medicine[Title/Abstract/Keywords] OR acupuncture[Title/Abstract/Keywords] OR massage[Title/Abstract/Keywords] OR aromatherapy[Title/Abstract/Keywords] OR bath therapy[Title/Abstract/Keywords] OR specific allergen immunotherap[Title/Abstract/Keywords] OR traditional healing[Title/Abstract/Keywords] OR naturopathy[Title/Abstract/Keywords] OR Ayurveda[Title/Abstract/Keywords] OR vitamins[Title/Abstract/Keywords] OR borage oil[Title/Abstract/Keywords] OR oral evening primrose oil[Title/Abstract/Keywords] OR meditation[Title/Abstract/Keywords] OR mindfulness[Title/Abstract/Keywords] OR psychological interventions[Title/Abstract/Keywords] OR prebiotics[Title/Abstract/Keywords] OR diet[Title/Abstract/Keywords] OR dietary supplements[Title/Abstract/Keywords] OR probiotics[Title/Abstract/Keywords] OR traditional medicine[Title/Abstract/Keywords]  #2. atopic eczema[Title/Abstract/Keywords] OR atopic dermatitis[Title/Abstract/Keywords]  #3. randomized controlled trial[Title/Abstract/Keywords] OR clinical trial[Title/Abstract/Keywords] OR blinding[Title/Abstract/Keywords] OR placebo[Title/Abstract/Keywords] OR random[Title/Abstract/Keywords]  #4. #1 AND #2 AND # 3 | |
| China National Knowledge Infrastructure (CNKI), and modified for the other three Chinese databases | 20/05/2018 |
| #1. shizhe (eczema) OR teyingxingpiyan (atopic dermatitis)  #2. zhongyi (traditional Chinese medicine) OR zhongyao (Chineses medicine) OR zhongyiyao (traditional Chinese medicine) OR zhongchengyao (Chinese patent medicine) OR caoyao (herb) OR bencao (medicine) OR minzuyao (ethnodrug) OR minjian (folk) OR zhenjiu (acupuncture and moxibustion) OR zhen (acupuncture) OR jiu (moxibustion) OR tuina (Tuina) OR anmo (massage) OR baguan (cupping) OR xuewei (acupoint) OR qigong (Qigong) OR guasha (Gua Sha) OR yuliao (balneotherapy) OR shunshi (homeopathy) OR cuimian (hypnosis) OR fangxiang (aromatherapy) OR fangsong (relax) OR shiliao (dietary therapy) OR yishengjun (probiotics) OR xinli (psychotherapy)  #3. duizhao (Control)  #4. dongwu (animal) OR shu (mouse) OR quan (dog) OR tu (rabbit) OR xitongzongshu (systematic review) OR xitongpingjia (systematic review) OR meta  #5. #1 AND #2 AND # 3 NOT #4 | |

| The GREAT database | 20/05/2018 |
| --- | --- |
| #1. Randomized controlled trial[Any field] AND parallel[Any field] AND Any treatment[complementary therapies] | |

| CAM-QUEST | 20/05/2018 |
| --- | --- |
| #1. Skin-Eczema-Ayurveda/Homeopathy/Phytomedicine/TCM | |

| AMED, EMBASE via OVID | 20/05/2018 |
| --- | --- |
| #1. (atopic eczema OR atopic dermatitis).ab  #2. (randomized controlled OR clinical trial OR blinding OR placebo OR random).ab  #3. (complementary medicine OR traditional medicine OR alternative medicine OR Chinese medicine OR herbal medicine OR acupuncture OR massage OR aromatherapy OR bath therapy OR specific allergen immunotherapy OR traditional healing OR naturopathy OR Ayurveda OR vitamins OR borage oil OR oral evening primrose oil OR meditation OR mindfulness OR psychological interventions OR prebiotics OR dietary exclusions OR dietary supplements OR probiotics).ab  #4. #1 AND #2 AND # 3 | |

| EBSCO | 20/05/2018 |
| --- | --- |
| #1. AB (atopic eczema OR atopic dermatitis)  #2. AB (randomized controlled OR clinical trial OR blinding OR placebo OR random)  #3. AB (complementary medicine OR traditional medicine OR alternative medicine OR Chinese medicine OR herbal medicine OR acupuncture OR massage OR aromatherapy OR bath therapy OR specific allergen immunotherapy OR traditional healing OR naturopathy OR Ayurveda OR vitamins OR borage oil OR oral evening primrose oil OR meditation OR mindfulness OR psychological interventions OR prebiotics OR dietary exclusions OR dietary supplements OR probiotics)  #4. #1 AND #2 AND # 3 | |
